# Bioprospecting Keratinous Materials

**DOI:** 10.4103/0974-7753.66915

**Published:** 2010

**Authors:** LN Jones, RD Sinclair, J Carver, H Ecroyd, Y Lui, LE Bennett

**Affiliations:** Department of Medicine, University of Melbourne, St Vincent’s Hospital, Melbourne 3065, Australia 5005; 1 School of Chemistry and Physics, University of Adelaide, Australia 5005,; 2 Food Science Australia, 3030, Australia 5005

**Keywords:** Bioactive peptides, bioprospecting, keratin

## Abstract

The concept of bioprospecting for bioactive peptides from keratin-containing materials such as wool, hair, skin and feathers presents an exciting opportunity for discovery of novel functional food ingredients and nutraceuticals, while value-adding to cheap and plentiful natural sources. The published literature reports multiple examples of proline-rich peptides with productive bio-activity in models of human disease including tumour formation, hypertension control and Alzheimer’s disease. Bioactive peptides have been identified from food and other protein sources however the bioactivity of keratin-related proteins and peptides is largely unknown. Considering the high representation of proline-rich peptides among proven bioactive peptides, the proline-rich character of keratinous proteins supports current research. A selection of mammalian (cow epidermis, sheep wool) and avian (chicken feather) keratinous materials were subjected to enzymatic hydrolysis using established processing methods. A bio-assay of determining inhibition of early stage amyloid aggregation involved using a model fibril-forming protein – reduced and carboxymethylated bovine K-casein (RCMk-CN) and quantitation of fibril development with the amyloid-specific fluorophore, Thioflavin T (ThT). The assay was fully validated for analytical repeatability and used together with appropriate positive controls. Peptide library products derived from chicken feather (n=9), sheep wool (n=9) and bovine epidermis (n=9) were screened in the fibril inhibition assay based on K-casein. 3 of 27 products exhibited interesting levels of bio-activity with regard to fibril inhibition. HPLC profiles provide an indication of the complexity of the assemblage of peptides in the three active products. We conclude the bioprospecting research using keratinous materials shows promise for discovery of useful bioactive peptides.

## INTRODUCTION

Proline-rich peptides are an important ingredient of a balanced human diet. The modern Western diet is often lacking in these peptides. While dairy products are a major traditional source of these peptides, other low-cost potential sources for dietary supplementation are desirable.

The concept of “bioprospecting” for bioactive peptides from keratin-containing materials such as wool, hair, skin and feathers presents an exciting opportunity for discovery and value-addition. The published literature (scientific and patent) reports multiple examples of proline-rich peptides with protective bioactivity in models of human disease, including tumor formation, hypertension control and Alzheimer’s disease. Bioactive peptides have been identified from food and other protein sources. However, the bioactivity of keratin-related proteins and peptides is largely unknown.

Considering the high representation of proline-rich peptides among proven bioactive peptides [Table 1], the proline-rich character of keratinous proteins supports their focus for biodiscovery research.

**Table 1:** Selection of known proline-rich peptides and related bioactives

Disease state	Substrate and bioactive peptide	Activity	Reference
Hypertension	Bovine casein:VPP, IPP	Angiotensin converting enzyme (ACE) inhibitor	Mizuno*et al.* (2004)
Alzheimer’s disease	Sheep colostrum: ‘Clostrinin ^TM^’	Beta sheet breaker (amyloid fibrils)	Inglot *et al.*(1996)
Colo-rectal cancer	Epithelial cellular proline-rich acidic protein	Epithelial cell growth homeostasis	Zhang *et al.* (2003)

## MATERIALS AND METHODS

A selection of mammalian (cow epidermis, sheep wool) and avian (chicken feather) keratinous materials were subjected to enzymatic hydrolysis using established processing methods for library production.

**Figure 1:**
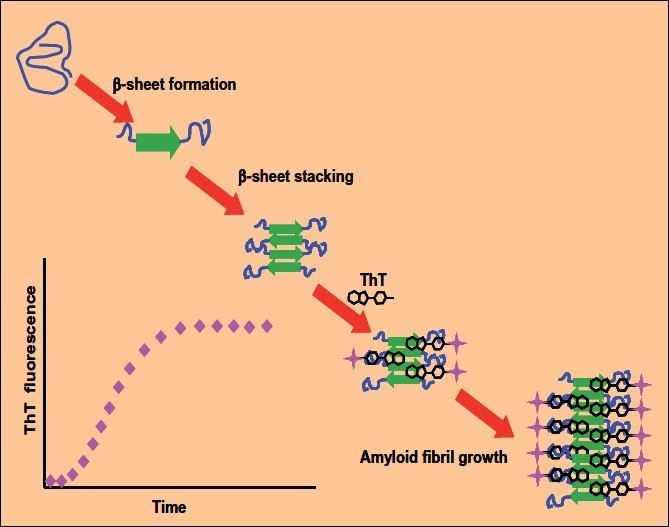
Schematic representation of the principle of the ThT fluorescence-based assay for monitoring assembly of beta-sheet structures associated with amyloid fibril growth

A bioassay for determining inhibition of early-stage amyloid aggregation involved using a model fibril-forming protein (reduced and carboxymethylated bovine kappa-casein, RCM kappa-CN) and quantification of fibril development with the amyloid-specific fluorophore, Thioflavin T (ThT, see Figure 1). Progressive aggregation of RCK kappa-CN was routinely monitored over 1,000 min by changes in fluorescence (440 nm excitation, 490 nm emission). The assay was fully validated for analytical repeatability and used together with appropriate positive controls.

## RESULTS AND DISCUSSION

**Figure 2:**
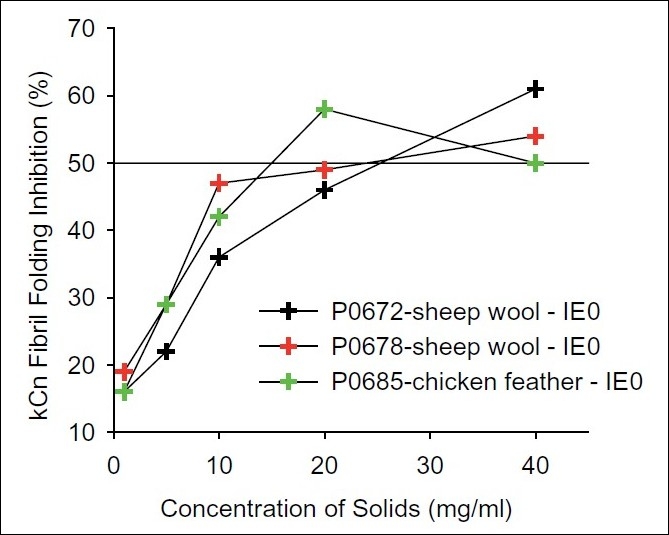
Dose-dependent inhibition of kappa-casein fibril assembly by peptide extracts

Peptide library products derived from chicken feather (*n*=9), sheep wool (*n*=9) and bovine epidermis (*n*=9) were screened in the fibril inhibition assay based on kappa-casein. Twenty-seven products were identified; three exhibited sufficient bioactivity to warrant enrichment and identification of active peptides within this sample set [Figure 2].

Results from the primary screening of keratinous peptide libraries in assays related to hypertension, including ACE inhibition and angiotensin II receptor inhibition, also indicate promising bioactivity. As fibril inhibition requires stoichiometric interaction between peptide and kappa-CN, the bioactivity is likely to be associated with most abundant peptides.

**Figure 3:**
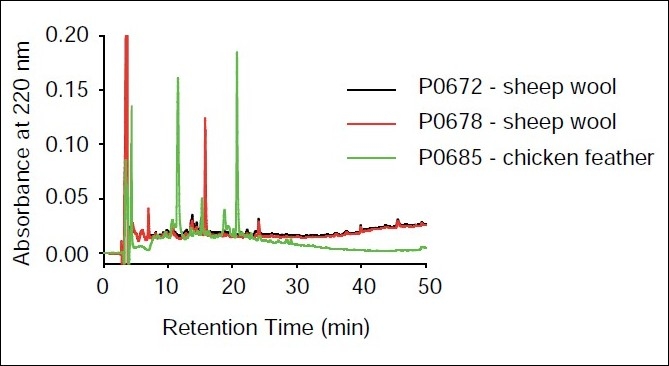
Phase high-performance liquid chromatography fingerprint profiles of lead antifibril products

High-performance liquid chromatography was used to provide an indication of the complexity of the assemblage of peptides in the three active products [Figure 3].

## CONCLUSIONS

Keratinous containing substances such as feather, wool and skin, often discarded after animal slaughter, can now be considered as important future neutraceuticals.

Furthermore, the model systems currently used allow characterization of the molecular properties of fibril-inhibiting peptides and will allow us to elucidate the mechanism and molecular properties of the keratinous peptides responsible for fibril inhibition. Bioprospecting research using keratinous materials facilitates the discovery of useful bioactives in research and product development.
